# Combined Minimally Invasive Transforaminal Lumbar Interbody Fusion and Minimally Invasive Laminectomy

**DOI:** 10.7759/cureus.115650

**Published:** 2026-09-02

**Authors:** Katherine Bunch, Simon Ammanuel, Bradley Schmidt

**Affiliations:** 1 Neurological Surgery, University of Wisconsin Hospitals and Clinics, Madison, USA; 2 Neurological Surgery, University of Wisconsin, Madison, USA; 3 Neurological Surgery, University of Wisconsin School of Medicine and Public Health, Madison, USA

**Keywords:** case instruction, interbody, laminectomy, minimally invasive, spine, transforaminal

## Abstract

Transforaminal lumbar interbody fusion (TLIF) is a well-established intervention for patients with spondylolisthesis and instability with compression of neural structures. Minimally invasive (MIS) TLIF has been proposed as potentially superior to a standard midline approach. For patients with both bilateral lateral recess stenosis and central stenosis, combining MIS-TLIF with MIS laminectomy allows for bilateral decompression, which is not usually afforded by standard TLIF, in addition to arthrodesis. This paper describes our technique for combining MIS-TLIF with MIS laminectomy, preserving midline structures and accomplishing decompression and arthrodesis via a unilateral approach. The use of CT-guided navigation in our technique significantly decreases radiation exposure to the surgical team compared with standard fluoroscopic techniques. Surgeons may consider this technique when treating patients with lumbar spondylolisthesis and instability who desire preservation of midline structures.

## Introduction

Decompression and fusion via transforaminal lumbar interbody fusion (TLIF) have historically been performed through a midline incision using an open procedure. Some have advocated for minimally invasive (MIS) technique alternatives, citing similar efficacy with the added benefits of shorter case durations, decreased blood loss, and fewer postoperative complications [1,2]. MIS laminectomy has been described as allowing central and lateral recess decompression via a muscle-sparing approach. This option has been associated with shorter hospital length of stay, less blood loss, fewer surgical site infections, and fewer major complications than open laminectomy [3,4]. While some papers discuss the basic methods and benefits of MIS-TLIF and MIS laminectomy, older techniques rely on fluoroscopy and do not allow bilateral decompression via a unilateral paramedian approach. This is due to the nature of fluoroscopy, which provides 2D visualization of the surgical field, as well as difficulties in assessing the extent of bilateral decompression and a steep learning curve for new surgeons. Currently, with advances in intraoperative computed tomography and neuronavigation, surgeons have a three-dimensional view of the surgical area and can obtain real-time guidance for surgical decompression, which can be performed with less radiation exposure compared with fluoroscopy. Our study describes our approach to achieving combined MIS-TLIF and MIS laminectomy, enabling bilateral decompression and fusion through a small, off-midline, unilateral approach using CT-based navigation.

## Technical report

For a single-level MIS-TLIF/laminectomy case, the authors select patients with spondylolisthesis and/or concern for instability at the index level with concurrent severe bilateral lateral recess stenosis. Informed consent is obtained. Patients are placed in a prone position on the spine table with the legs in anatomic position. A reference frame is placed in the iliac crest through a percutaneous incision. The optimal location of this reference frame is the caudal portion of the posterior superior iliac spine (PSIS) (Figure 1).

**Figure 1:**
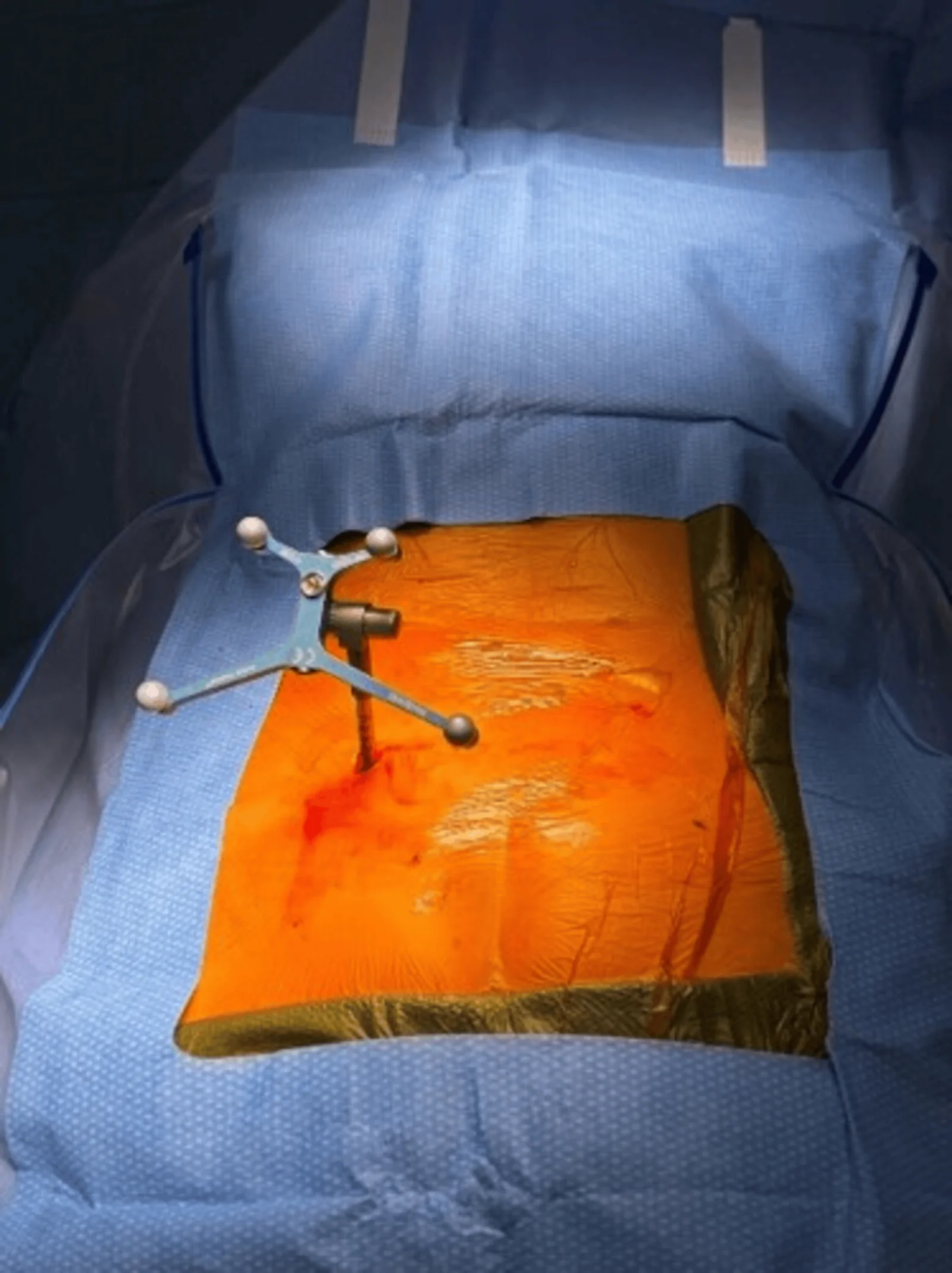
Docking of the navigation reference frame onto the PSIS. PSIS: Posterior superior iliac spine.

Intraoperative CT is used to obtain images for computerized stereotactic navigation, which is subsequently registered to the patient’s anatomy. We then use navigation to localize our off-midline incisions (Figure 2).

**Figure 2:**
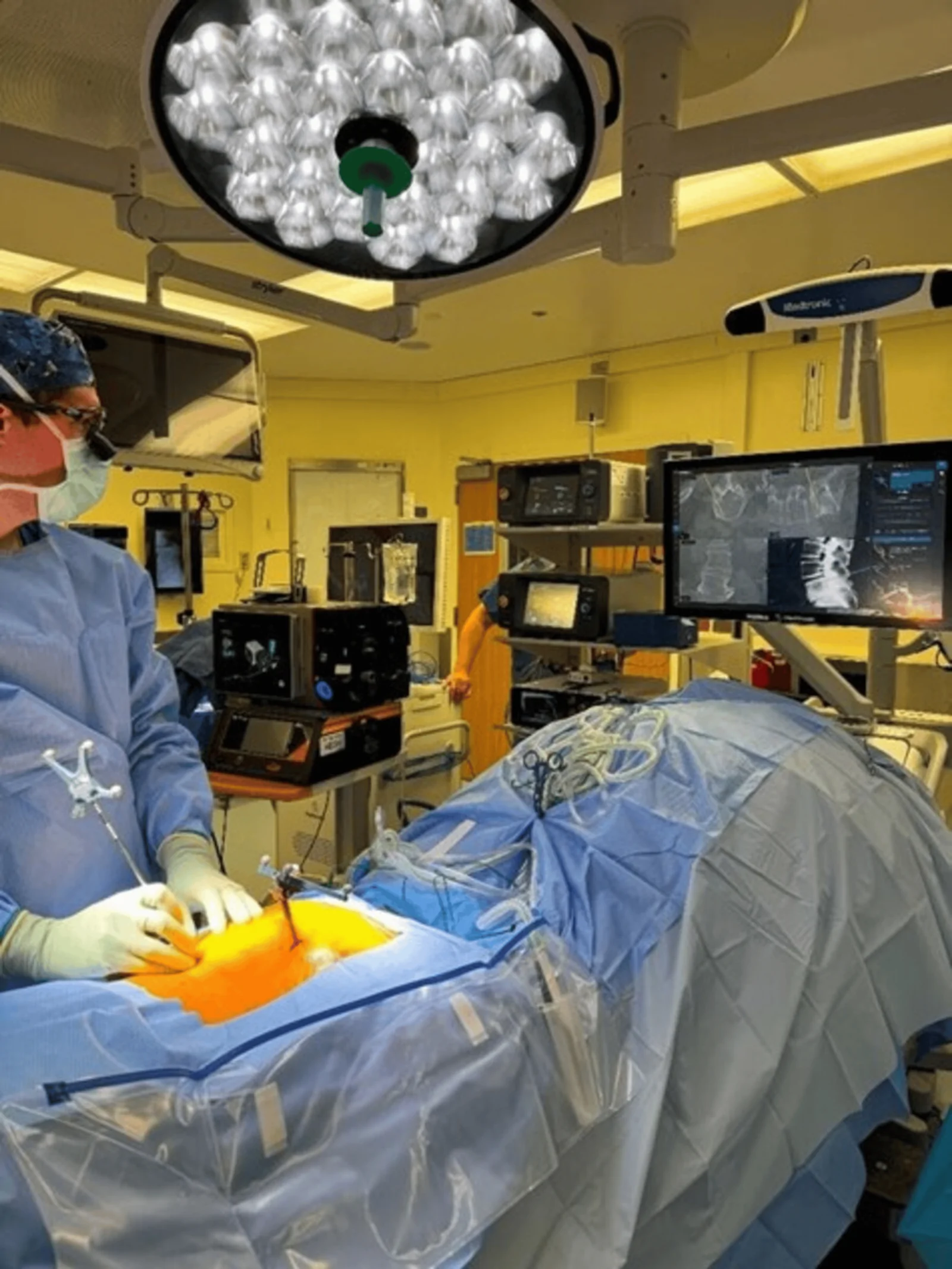
Planning of off-midline incisions.

Navigation-assisted pedicle screws are inserted through percutaneous stab incisions at the appropriate levels. Dissection of the lateral border of the multifidus muscle on the side on which the TLIF will be performed is critical, as it allows for a clean plane for the retractor system, lower blood loss, and preservation of the multifidus attachments along the spinous processes.

We then attach our MIS retractor/distractor and begin the MIS-TLIF. The surgical microscope is brought into the field. The corresponding facet and lateral lamina are exposed. Using a combination of an ultrasonic bone cutter and rongeurs, a complete facetectomy is performed. The facetectomy is optimally performed with the ultrasonic bone cutter using three simple cuts: a cut along the ipsilateral spinolaminar line, a cut along the pars of the rostral vertebra, and a cut along the superior articular process (SAP) of the caudal vertebra (Figure 3).

**Figure 3:**
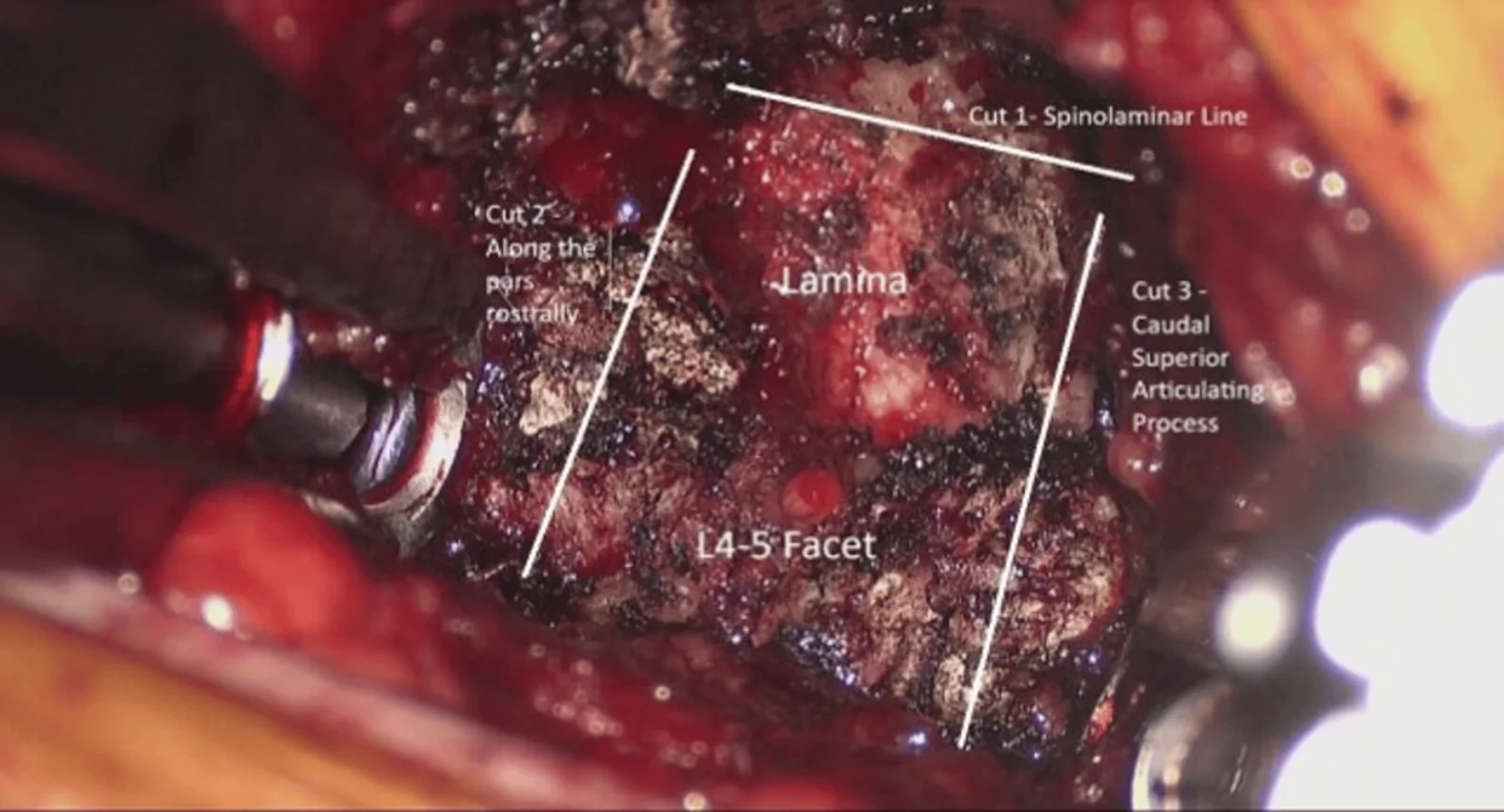
Planned facetectomy with laminectomy. Facetectomy using three simple cuts: (1) a cut along the ipsilateral spinolaminar line, (2) a cut along the pars of the rostral vertebra, and (3) a cut along the superior articular process of the caudal vertebra.

The entire facet may then be removed in one piece, allowing it to be morselized for interbody autograft. Next, the bilateral decompressive laminectomy and medial facetectomy are completed. The medial blade of the retractor system is slightly raised, and the patient is “airplaned” away from the operative side. The spinous process is then undermined and drilled down, initially leaving the ligamentum flavum to serve as a protective layer. The medial facet and ligamentum flavum are then resected to decompress the central canal and lateral recesses (Figure 4).

**Figure 4:**
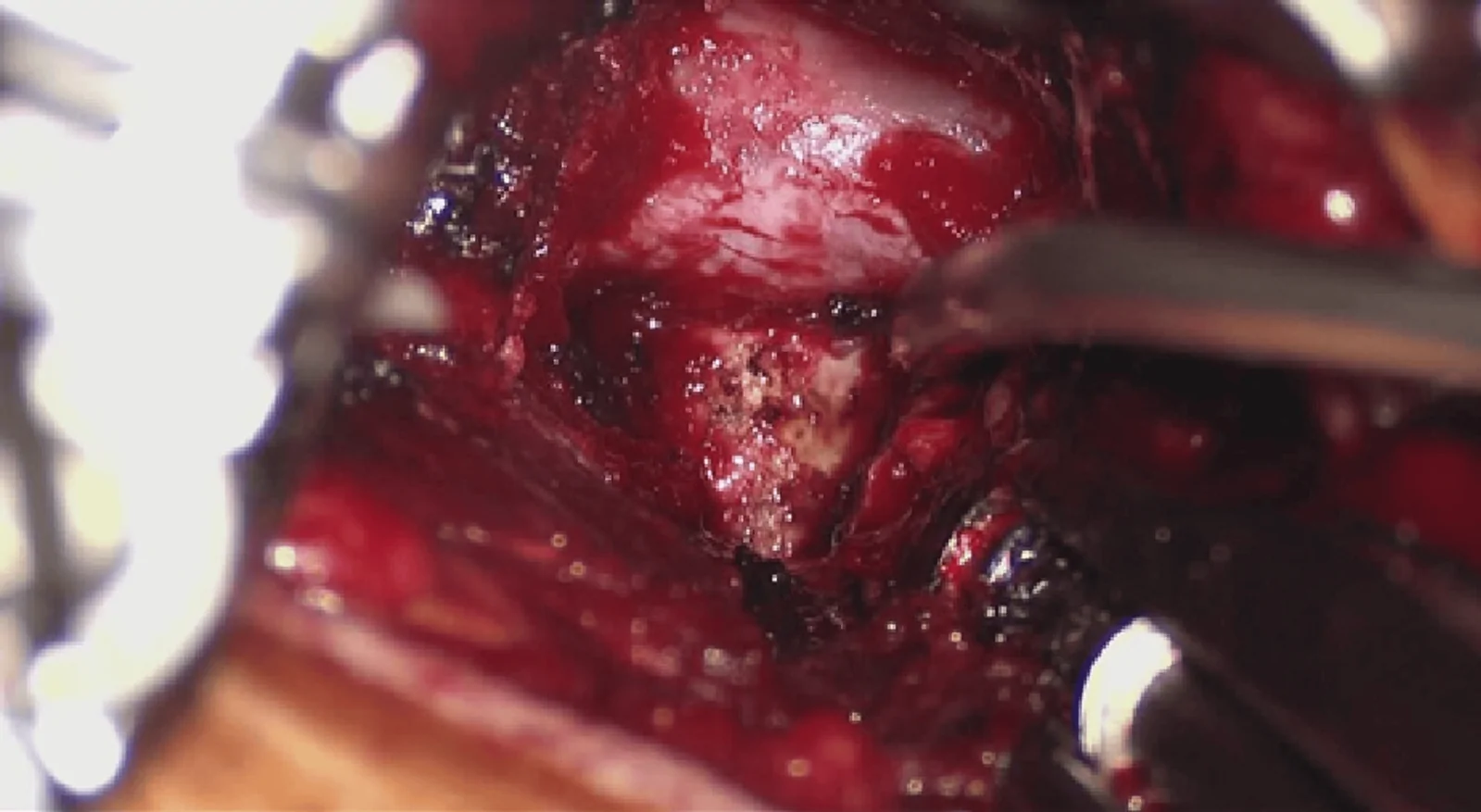
Exposure of the thecal sac after facetectomy and laminectomy. Decompression of the central canal and bilateral lateral recesses is completed.

The navigated probe can be used to confirm that the midline has been crossed and that the contralateral lateral recess is decompressed (Figure 5).

**Figure 5:**
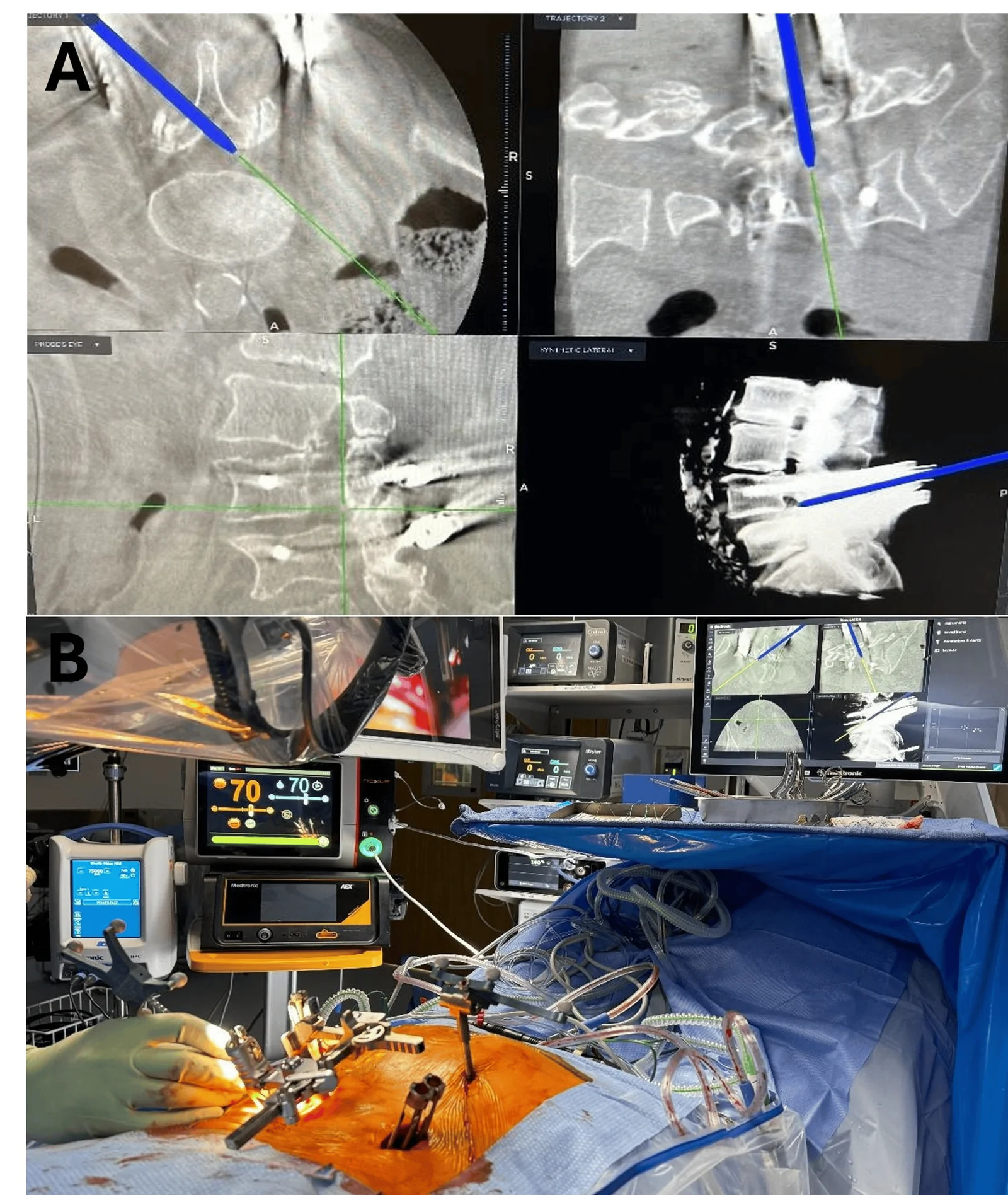
Navigated view of bilateral lateral recess decompression via an off-midline approach. (A) The navigated probe shows contralateral decompression. (B) Live view of the probe being placed in the contralateral recess.

We then proceed with a complete discectomy and endplate preparation. Intervertebral biomechanical devices are placed. Morselized bone graft and allograft are packed into the vertebral interspace for interbody arthrodesis. The preferred cage used by the author allows for expansion once in the disc space, which can greatly improve focal lordosis and correction of spondylolisthesis at the target level.

The rods are placed bilaterally via standard percutaneous techniques. Cap screws are placed and tightened. Post-instrumentation imaging is obtained. The wounds are irrigated, hemostasis is achieved, and the wounds are closed in a layered fashion. The incisions are no longer than 5-6 cm in length (Figure 6).

**Figure 6:**
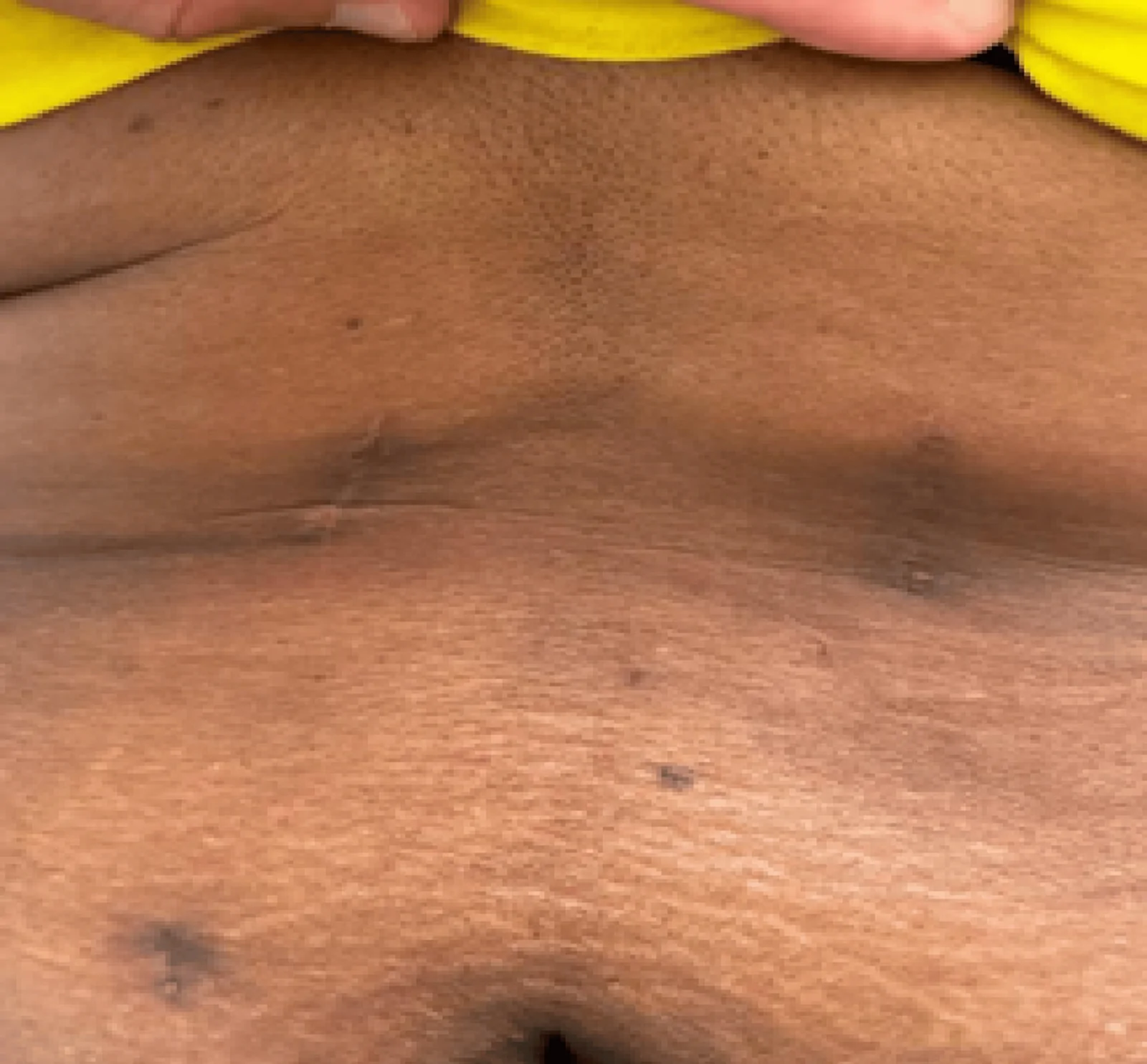
Healed MIS incisions. MIS: Minimally invasive surgery.

There is almost no muscle retraction, as natural muscle planes are utilized. We have not had any unanticipated events with our combination of these techniques.

## Discussion

While there has yet to be a randomized controlled trial comparing TLIF with MIS-TLIF, studies have demonstrated equivalent to improved patient and radiographic outcomes, as well as lower reoperation rates, with MIS-TLIF compared with TLIF [1,2,4,5]. These studies did not combine MIS-TLIF with MIS laminectomy and do not comment on the increased spinal canal diameter afforded by combining the procedures. Meta-analyses have shown favorable outcomes with MIS laminectomy, including decreased hospital length of stay (LOS), reduced blood loss, and fewer surgical site infections [4,5]. While not necessary in every TLIF, the addition of MIS laminectomy can allow notable increases in canal diameter (Figure 7).

**Figure 7:**
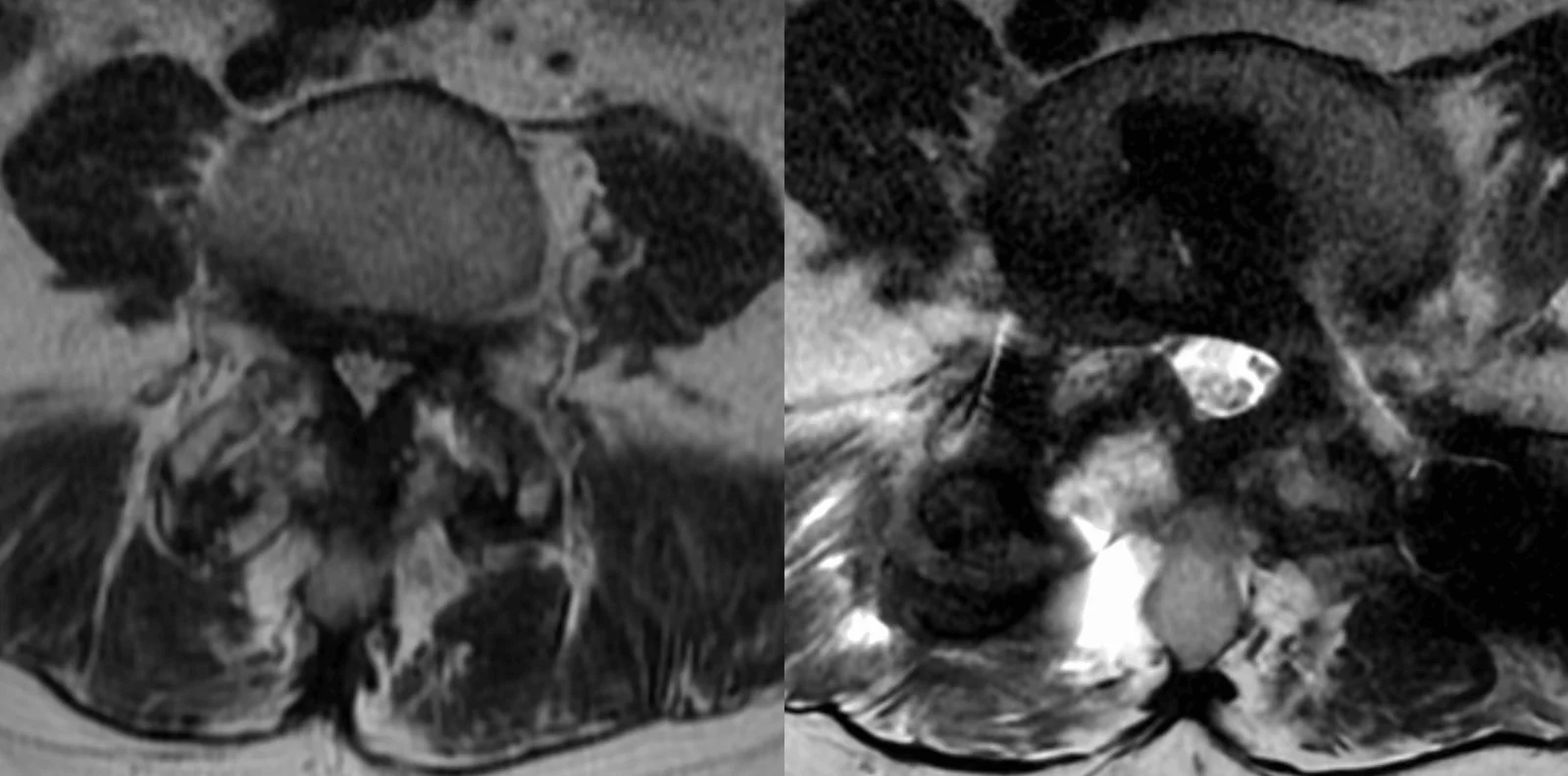
Preoperative and postoperative MRI following the combined MIS technique, demonstrating increased spinal canal diameter postoperatively. MIS: Minimally invasive surgery.

In developing this technique, we encountered patients with concurrent lumbar spinal stenosis and mobile spondylolisthesis, for whom the standard of care is posterolateral fusion with midline laminectomy via an open midline approach. We sought to apply MIS techniques to reduce complication rates compared with standard open surgery. For appropriate patients, the combined MIS technique allows greater decompression than would normally be achievable with a unilateral standard approach. With the advantages of CT-based navigation and advancements in retractor systems, surgeons can obtain wide exposure of the surgical anatomy through a smaller incision and achieve better assessment of bilateral decompression during laminectomy with real-time feedback.

Use of intraoperative CT in our technique eliminates the need for conventional fluoroscopy, thereby reducing procedural radiation exposure to the surgical team. Wong noted a radiation exposure of 1.90 mSv in the MIS-TLIF group (completed with fluoroscopy) compared with 0.75 mSv in the standard TLIF group [6]. Wong and colleagues estimated 273.6 mSv over 144 MIS-TLIFs, associated with a 2.7% increased lifetime risk of cancer [6]. The radiation risk to the surgeon is significantly reduced with the use of intraoperative CT, as operators can step to a radiation-free location during image acquisition. A standard case using intraoperative CT is estimated to deliver a patient dose of 14.3 mSv, which is roughly equivalent to that of a lumbar spine CT (14.6 mSv) [6]. This radiation dose to the patient is higher than that with fluoroscopy, but not prohibitively so.

MIS techniques in spine surgery are continuously being developed. There is no strictly established definition of what constitutes “minimally invasive” surgery or what exactly defines an “MIS-TLIF.” Given that no single, strictly defined operative technique exists, authors must disclose their operative techniques, as outcomes may not be generalizable if techniques differ significantly. Our technique contributes to the discourse on what is possible through MIS approaches.

## Conclusions

MIS-TLIF is a safe and effective procedure that provides outcomes at least equivalent to those of standard TLIF. This technical report discusses a combined technique of MIS-TLIF and MIS laminectomy that allows decompression and deformity correction in patients with grade I spondylolisthesis and concurrent bilateral lateral recess stenosis, with the benefits of MIS including decreased blood loss and operative time. Our technique uses intraoperative CT combined with navigation to reduce staff radiation exposure and provide a 3D view of the surgical anatomy compared with fluoroscopy. This technical report provides surgeons with a step-by-step process to achieve adequate decompression and correction.
